# Current status and perspectives of spread through air spaces in lung cancer

**DOI:** 10.1111/1759-7714.13918

**Published:** 2021-05-05

**Authors:** Toshihiro Ikeda, Kyuichi Kadota, Tetsuhiko Go, Reiji Haba, Hiroyasu Yokomise

**Affiliations:** ^1^ Department of General Thoracic Surgery, Breast and Endocrinological Surgery, Faculty of Medicine Kagawa University Takamatsu Japan; ^2^ Department of Diagnostic Pathology, Faculty of Medicine Kagawa University Takamatsu Japan

**Keywords:** invasion, lung cancer, recurrence, spread through air spaces, STAS

## Abstract

According to the World Health Organization classification of 2015, spread through air spaces (STAS) is a newly recognized pattern of invasion in lung adenocarcinoma. Many researchers have reported that STAS is recognized in all histological subtypes, and there is a strong association between STAS and prognosis in lung cancer. However, there are several technical issues associated with STAS, such as distinction between the actual in vivo phenomenon and an artifact, difficulty in assessing STAS in frozen specimens, and establishing the relationship between morphological and molecular properties of STAS. This review focuses on the current state of knowledge and the outlook of the STAS phenomenon from the perspective of surgeons, pathologists, and radiologists.

## Graphical abstract

There have been previous reports in the literature which have investigated the frequency of STAS, histological subtypes, prognostic factors, expression of various markers by immunostaining, gene mutation status, and PD‐L1 association with STAS. This review indicates that more evidence is required to optimize STAS classification and treatment decisions for STAS‐positive patients.

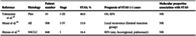

## INTRODUCTION

Currently, lung cancer is the most prevalent disease, and has the highest mortality of all malignant neoplasms.^1^ In addition, despite early detection through development of imaging technology, lung cancer has maintained a high mortality rate due to high recurrence. Invasion in lung cancer has been defined as: (i) the presence of nonlepidic patterns such as acinar, papillary, solid or micropapillary patterns; (ii) infiltration of stroma; and (iii) lymphatic and vascular invasion or infiltration of structures such as the visceral pleura.^2^ The effect of invasion on recurrence and prognosis has been proven. Spread through air spaces (STAS) is a newly recognized pattern of invasion previously described by the World Health Organization (WHO) in 2015.^3^ Since 2015, several reports have been published on STAS, and have attracted the attention of clinicians involved in the treatment of lung cancer. This review aimed to highlight the current knowledge on the STAS phenomenon from the perspective of surgeons, pathologists, and radiologists.

## HISTORY

**FIGURE 1:**
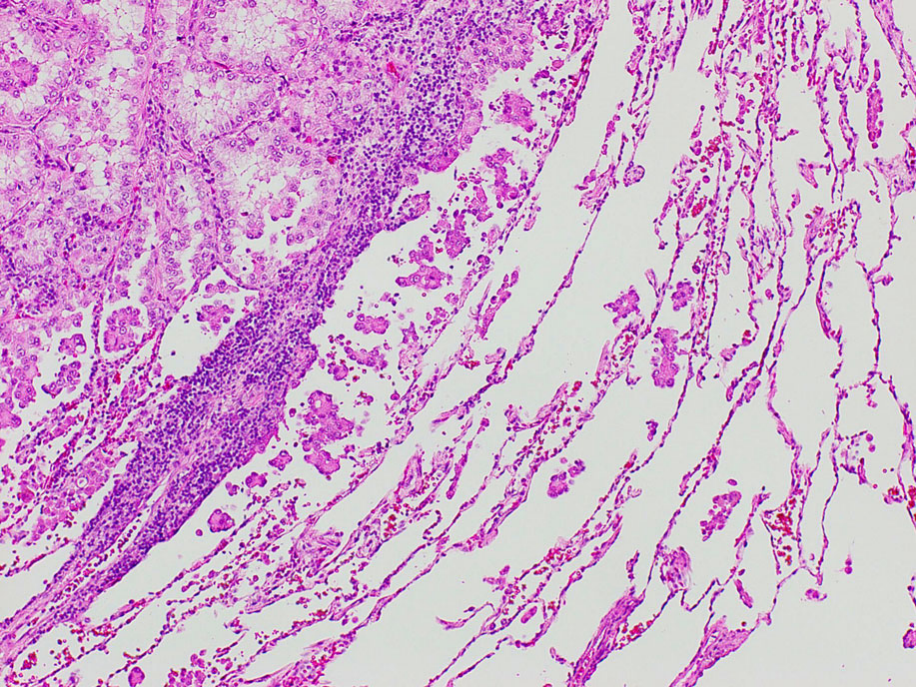
In 2015, the concept of spread through air spaces (STAS) was described by the World Health Organization (WHO) as consisting of micropapillary clusters, solid nests, or single cells identified beyond the edge of the tumor invading into the air spaces surrounding the lung parenchyma

Since 1995, pathologists have known that pathological sections of lung cancer may show “aerogenous spread.” However, at that time, “aerogenous spread” was a recognized pattern of spread of lung cancer.^4^ Through the studies on the pathological characteristics of lung adenocarcinoma, Amin et al.^5^ reported that the micropapillary component in lung adenocarcinoma, defined as small papillary clusters of glandular cells growing within an air space, was prone to recurrence. In 2011, the International Association for the Study of Lung Cancer / American Thoracic Society / European Respiratory Society defined five major histological patterns and four variants of lung adenocarcinoma. According to this classification, micropapillary predominant lung adenocarcinoma was associated with a poor prognosis.^2^ In 2013, Onozato et al.^6^, ^7^ proposed the term “tumor islands,” that referred to a large collection of isolated tumor cells within alveolar spaces. In this study, although tumor islands demonstrated continuity from the primary lesion by three‐dimensional reconstruction, they were significantly associated with a worse recurrence‐free survival (RFS). In 2015, the concept of “STAS” was described by the WHO as consisting of micropapillary clusters, solid nests, or single cells identified beyond the edge of the tumor invading into the air spaces surrounding the lung parenchyma (Figure 1).^3^ According to the WHO classification, STAS is not included in the percentage measurement of subtype patterns or the size of tumor invasion, and is considered to be a pattern of invasion similar to visceral pleural and vascular invasion. Furthermore, minimally invasive adenocarcinoma and adenocarcinoma in situ are defined as having no STAS. As STAS is a relatively new pattern of lung cancer invasion, numerous aspects of the entity remain unclear.

## ASSOCIATION BETWEEN STAS AND PROGNOSIS IN LUNG CANCER

In 2015, Kadota and Travis et al.^8^ reported for the first time that in the limited resection group, the cumulative incidence of recurrence (CIR) for both distant and locoregional recurrence was significantly higher in patients with STAS‐positive tumors than in those with STAS‐negative tumors. Subsequently, the relationship between STAS and overall survival (OS) and RFS in lung cancer of various stages and histological types was reported. Previous studies that focused on STAS in lung cancer are shown in Table 1.^9^, ^10^, ^11^, ^12^, ^13^, ^14^, ^15^, ^16^, ^17^, ^18^, ^19^, ^20^, ^21^, ^22^, ^23^, ^24^, ^25^, ^26^, ^27^, ^28^, ^29^, ^30^, ^31^ Many researchers reported that there were approximately 15%–60% STAS‐positive cases, and the more advanced the disease stage, the higher the rate of STAS‐positivity. Many reports showed that patients with STAS‐positive tumors were associated with significantly reduced RFS and OS; in particular, there is a strong association between recurrence and STAS. Although the percentage of recognition of STAS differs depending on the histological types of lung cancer, the presence of STAS has already been reported in adenocarcinoma, squamous cell carcinoma, small cell carcinoma (SCLC), lung neuroendocrine tumors (NETs), and pleomorphic cancer, and STAS may be expressed in all lung cancer histological types. In addition, STAS is more commonly observed in lung cancer with highly malignant histological characteristics such as solid components, micropapillary components, and cribriform patterns.^20^, ^32^ There are three meta‐analysis reports based on these results.^33^, ^34^, ^35^ Wang et al.^33^ pooled the data of 3231 patients from eight studies and reported that STAS was associated with a poor OS (HR = 1.49, 95% CI: 1.29–1.72) and RFS (HR = 1.79, 95% CI: 1.57–2.04) in non‐small cell lung cancer (NSCLC). Similarly in a meta‐analysis by Liu et al.,^34^ STAS was an independent negative prognostic factor for OS (HR = 1.78, 95% CI: 1.51–2.11) and RFS (HR = 1.84, 95% CI: 1.59–2.12) in 12 studies with 3564 NSCLC patients. Chen et al.^35^ also reported a poorer OS (HR = 1.75, 95% CI: 1.38–2.23) and RFS (HR = 1.98, 95%CI; 1.69–2.31) in 14 studies with 3754 NSCLC patients; subgroup analysis by histological types indicated that the presence of STS was significantly associated with inferior OS (HR = 1.96, 95% CI: 1.47–2.61) and RFS (HR = 2.29, 95% CI: 1.84–2.84) in lung adenocarcinoma. Therefore, STAS has a significant impact on prognosis at any stage and histological type of lung cancer.

**TABLE 1:** Previous studies that investigated the frequency of STAS, histological subtypes, prognostic factors, expression of various markers by immunostaining, gene mutation status, and PD‐L1 association with STAS

Reference	Histology	Patient number	Stage	STAS, %	Prognosis of STAS (+) cases	Molecular properties association with STAS
Kadota et al.^8^	Ad	411	I	38.0	CIR (any, distant, locoregional) (limited resection group)	NR
Warth et al.^9^	Ad	569	I–IV	50.6 Limited: 21.6 Extensive: 29.0	OS, DFS	*EGFR* wild‐type BRAF NS (KRAS)
Shiono et al.^10^	Ad	318	I	14.8	OS, RFS	*EGFR* wild‐type
Dai et al.^11^	Ad	544	I (size < 3 cm)	30.3	OS, RFS	NR
Uruga et al.^12^	Ad	208	I (size < 2 cm)	47.6 Low STAS: 18.3 High STAS: 29.3	OS, RFS	NR
Toyokawa et al.^13^	Ad	327	I	58.4	OS, RFS	NS (*EGFR* mutation, PD‐L1)
Kim et al^14^	Ad	276	I–III	33.0	RFS	ALK (+)
Toyokawa et al.^15^	Ad	276	I	55.4 Low STAS: 17.4 High STAS: 38.0	OS, RFS	NS (PD‐L1)
Lee et al.^16^	Ad	316	I–III	50.6	OS, RFS	*EGFR* wild‐type ALK (+) ROS1 (+) NS (KRAS)
Liu et al.^17^	Ad	208	I–III	51.4	OS, RFS	MTA1
Hu et al.^18^	Ad	500	I–III	26.8	NR	*EGFR* mutation KRAS (−) BRAF (−) *HER2* wild‐type ALK (+)
Eguchi et al.^19^	Ad	1497	I (T1N0M0)	40.5	OS, LC‐CID, CIR	NR
Kadota et al.^20^	Ad	735	I–IV	33.6	(only stage I) OS RFS (any, locoregional)	ALK (+)
Ren et al.^21^	Ad	752	IA	28.7	OR RFS	NR
Terada et al.^22^	Ad	76	III (N2)	60.5	RFS	NR
Lu et al.^23^	Sq	445	I–III	29.7 Limited:7.2 Extensive: 22.5	LC‐CID CIR (any, distant, locoregional)	NR
Kadota et al.^24^	Sq	216	I–IV	40.0	RFS (any, distant, locoregional)	NR
Yanagawa et al.^25^	Sq	220	I–III	19.1	OS (stage I) RFS (stage I)	NR
Toyokawa et al.^26^	SCLC	30	I–IV	83.0	No significance	NR
Jia et al.^27^	Ad, Sq	424	I–IV	Ad: 60.4 Sq: 32.2	RFS, OS (Ad)	Low E‐cadherin expression High vimentin expression High survivin expression (only Ad)
Aly et al.^28^	NETs	487	I–IV	26.0 LCNEC: 43.0 SCLC: 46.0	LC‐CID (LCNEC, SCLC) CIR (LCNEC)	NR
Yokoyama et al.^29^	Pleo	35	I‐III	40.0	OS, RFS	NR
Masai et al.^30^	All	508	I‐IV	15.0	Local recurrence (limited resection group)	NR
Shiono et al.^31^	NSCLC	848	I	16.4	RFS (any, locoregional, pulmonary)	NR

Abbreviations: Ad, adenocarcinoma; ALK, anaplastic lymphoma kinase rearrangement; BRAF, v‐raf murine sarcoma viral oncogene homolog B1 mutation; CIR, cumulative incidence of recurrence; DFS, disease‐free survival; EGFR, epidermal growth factor receptor mutation; HER2, human epidermal growth factor receptor type 2 mutation; KRAS, kirsten rat sarcoma viral oncogene homolog mutation; LC‐CID, lung cancer–specific cumulative incidence of death; MTA1, metastasis‐related protein 1.; NETs, lung neuroendocrine tumors; NR, not reported; NS, not significant; NSCLC, non‐small cell lung cancer; OS, overall survival; PD‐L1, programmed cell death‐1 ligand; Pleo, pleomorphic carcinoma; RFS, recurrence‐free survival; ROS1, c‐ros oncogene 1 rearrangement; SCLC, small cell lung cancer; Sq, squamous cell carcinoma; STAS (+), STAS‐positive tumor.

## QUANTITATIVE ASSESSMENT OF STAS

The quantitative evaluation of STAS is an issue of debate among pathologists. Uruga et al.^12^ classified STAS into low STAS (1–4 single cells or clusters) and high STAS (≥5 single cells or clusters), and reported that increasing STAS number was associated with higher histopathological grade (solid predominant invasive adenocarcinoma), lymphatic invasion, pleural and vascular invasion, and larger tumor size. However, there was no significant difference in RFS between the groups with absence of and low STAS. Using the same classification, Toyokawa et al.^15^ demonstrated that STAS is an independent factor of OS and RFS; however, they could not demonstrate specific correlation depending on the number of STAS. Currently, there is no consensus on the quantitative assessment of STAS, and further its subdivision remains controversial.

## ASSOCIATION WITH SURGICAL PROCEDURE

The standard surgical procedure for early stage NSCLC is anatomic lobectomy and lymph node dissection^36^; however, sublobar resection including segmentectomy or wedge resection may be an option depending on the tumor‐node‐metastasis classification stage and general condition of the patient. Kadota et al.^8^ first reported the relationship between STAS and surgical procedures in a retrospective cohort of 411 small (<2 cm) resected stage I adenocarcinomas. STAS was significantly correlated to distant and locoregional recurrence in the limited resection group; however, there was no association with recurrence in the lobectomy group. Following this report, several reports^20^, ^21^, ^31^, ^37^ demonstrated that sublobar resection conferred low RFS and OS in patients with STAS‐positive tumors. Liu et al.^34^ reported that in the lobectomy group, patients with STAS had a trend of shorter RFS than those without STAS (HR: 1.67, 95% CI: 0.93–2.68); in addition, in a subgroup analysis of a meta‐analysis that included 14 studies, the presence of STAS was associated with shorter RFS in those undergoing limited resection (HR: 4.05, 95% CI: 2.31–7.09). Eguchi et al.^19^ performed a propensity score‐matched analysis of 1497 patients and reported that in those with STAS‐positive T1N0M0 lung adenocarcinoma, the lobectomy group had better CIR (16% versus 39%) and CID‐LC (8% versus 16%) than the sublobar resection group. Interestingly, the author also reported that in STAS‐negative tumors, the sublobar resection group in which the surgical margin was sufficiently wide (surgical margin to tumor diameter ≥ 1) had lower locoregional recurrence than the group, in which the surgical margin was insufficiently wide (margin to tumor diameter ratio < 1). However, in the case of STAS‐positive tumors with sublobar resection, there was no significant difference between the groups with sufficiently and insufficiently wide surgical margins (16% versus 25%). This results indicate that the concept that the surgical margin for sublobar resection in early‐stage lung cancer should be equal to the tumor diameter^38^, ^39^, ^40^ may be inappropriate in the case of STAS‐positive tumors. Similarly, Masai et al.^30^ reported that after limited resection, the presence of STAS and tumor margins of less than 1.0 cm were significant risk factors for local recurrence, but not distant recurrence in early‐stage lung cancer. Locoregional recurrence is the most notable event after lung cancer surgery, and the two reports on surgical margins in STAS‐positive tumors are important to surgeons. Therefore, it is ideally desirable to accurately determine the presence or absence of STAS preoperatively or intraoperatively.

## PREOPERATIVE ASSESSMENT OF STAS

The presence or absence of STAS possibly affects the operative procedure and prognosis, but there is no report on its preoperative detection; surgery is the only method for evaluating STAS. An accurate prediction of preoperative STAS is important for treatment planning. Therefore, several reports have aimed to indirectly predict STAS preoperatively based on the imaging findings of lung cancer. Toyokawa et al.^13^ studied CT features in 327 cases of lung adenocarcinoma with surgical resections, and reported that the CT features associated with STAS‐positive tumors are the radiographic tumor diameter > 2.0, vascular convergence, negative‐surrounding ground‐glass opacity (GGO), notch, pleural indentation, spiculation on univariable analysis, and negative‐surrounding GGO and notch in multivariable analysis. In addition, the proportion of STAS‐positive tumors increased in the consolidation to tumor ratio (CTR). Kim et al.^14^ and Margerie‐Mellon et al.^41^ also found that the STAS‐positive tumors were associated with tumor solid component size and the presence of an abundant nonsolid component. Kim et al.^14^ defined a cutoff value of 90% for the percentage of the solid component and reported a sensitivity of 89.2% and a specificity of 60.3%. The author also reported that pure solid lesions showed three‐fold greater STAS‐positivity than part solid lesions (odds ratio, 3.27), and pure GGO or those with solid component percentages <40% did not show STAS‐positive tumors. The strong association between solid nodules and STAS‐positive tumors was consistent with the fact that STAS is associated with tumors of high pathological grade (micropapillary, cribriform, and solid adenocarcinoma), as reported by Kadota et al.^8^ and Warth et al.^9^ In addition, Kim et al.^14^ examined the maximum diameter of the solid component, and determined that the optimal cutoff value is 15 mm; however, the efficacy was low, with a sensitivity of 86.0% and a specificity of 45.1%. In the report by Toyokawa et al.,^13^ a significant difference was observed between STAS‐positive tumors of >2 cm and ≤2 cm; in addition, solid nodules > 2 cm were also associated with STAS‐positive tumors in the report by de Margerie‐Mellon et al.^41^ Yin et al. reported that there was no significant relationship between radiological tumor size > 2 cm and STAS (HR: 1.47, 95% CI: 0.86–2.51), but there was a significant relationship between the percentage of solid component >50% and STAS (HR: 2.95, 95% CI: 1.88–4.63) in the meta‐analysis. Shiono et al.^10^, ^37^ focused on the maximum standardized uptake value (SUV‐max) using FDG‐PET as an image support other than CT; however, they did not provide a specific opinion on the relationship between STAS and SUV‐max. Definite imaging findings that predict STAS presence before surgery have as yet not been identified; however, imaging findings suggestive of malignant characteristics such as the solid diameter and CTR have been associated with STAS‐positive tumors. Therefore, preoperative image evaluation is useful for prediction of the presence of STAS. It is hoped that future studies will find more powerful STAS predictive factors.

## INTRAOPERATIVE ASSESSMENT OF STAS (FROZEN SECTIONS)

As described above, the relationship between the presence or absence of STAS and the surgical procedure is clear, and confirming the presence of STAS greatly affects patient prognosis. Therefore, it is important to be able to accurately evaluate frozen sections during intraoperative rapid tissue diagnosis. Several studies have addressed this issue. Interestingly, one of them by Eguchi et al.^19^ reported that the sensitivity and specificity of STAS detection on frozen sections were 71% and 92%, respectively, suggesting that STAS can be recognized reliably using frozen sections. However, Walts et al.^42^ reported that the frozen section sensitivity in detecting STAS was only 50%, with 100% positive predictive and 8% negative predictive values. Furthermore, this study suggested that it was difficult to use intraoperative detection of STAS as a useful predictive feature for stratifying patients for either lobectomy or sublobar resections. Morimoto et al.^43^ indicated that that evaluation of STAS in frozen sections was difficult, because the resected lungs were not sufficiently inflated. It is currently difficult to determine the presence or absence of STAS during intraoperative rapid tissue diagnosis; more studies will therefore need to be conducted.

## DIFFERENCES BETWEEN STAS AND AN ARTIFACT

When assessing STAS in pathological specimens, it is necessary to distinguish between STAS as an in vivo phenomenon and an artifact. Thunnissen et al.^44^ reported that tumor cells may be displaced by the knife along the plane of sectioning; the phenomenon of floating tumor cells in alveolar spaces, that were created artificially during processing at the pathology laboratory was called “spreading through a knife surface” (STAKS). Artifacts created by displacement by the knife during tissue processing and slide preparation is reported to occur in 0.01%–2.9% of cases.^45^, ^46^ Blaauwgeers et al.^47^ reported that tumor islands or loose tumor cells are identified in 73% of cases and the majority may be attributed to mechanical artifacts related to surgical resection and gross room specimen processing. It is true that it is difficult to distinguish between STAKS and STAS, because there is no clear standardized method for processing the resected specimen and preparing pathological sections. However, Lu et al.^48^ reported on two cases of an extensive STAS predominant pattern, wherein the main tumor was not cut either by the surgeon or pathologist; this provides further evidence that STAS is not an artifact. Yagi et al.^49^ reported that STAS cells were focally attached to the alveolar walls, in a manner consistent with the concept of “co‐option” of the pre‐existing blood vessels. This fact may help distinguish between STAS and an artifact. Many researchers are involved in the assessment and treatment of STAS‐positive lung cancer, based on the premise that STAS is not merely an artifact. It is true that it is difficult to distinguish between STAS and an artifact, but many reports have confirmed that the former is associated with a poor prognosis, and cannot be treated as a mere artifact.

## ASSOCIATION WITH MOLECULAR PROPERTIES

One of the difficulties in the accurate assessment of STAS is that the relationship between morphological and molecular properties has not been fully clarified. Kadota et al.^23^ found that STAS was associated with tumor budding, which is known to be associated with vimentin expression; this is one of the markers of epithelial‐mesenchymal transition (EMT) related to cancer cell migration and invasion.^50^, ^51^ Therefore, Kadota et al.^24^ examined the association between vimentin expression and STAS. While the difference found was not significant, the expression of vimentin in STAS‐positive tumors tended to be higher than that in STAS‐negative tumors (48% versus 32%). Furthermore, no significant difference was observed in the downregulation of E‐cadherin, another EMT marker. However, Jia et al.^27^ reported that STAS was associated with low‐E‐cadherin expression, and high vimentin expression in adenocarcinoma and squamous cell carcinoma. Furthermore, Jin et al.^52^ reported that c‐ros oncogene 1 (*ROS1*)‐rearranged lung cancer showed frequent STAS‐like aerogenous spread manifested by a decrease in E‐cadherin levels; Lee et al.^16^ also reported that ROS‐1 was highly expressed at 71% of STAS‐positive tumors. In addition, Liu et al.^17^ examined the association of metastasis‐related protein 1 (MTA1), reported to be associated with high metastasis and poor prognosis by Li et al.^53^ This report showed that there is a significantly higher MTA‐*1* expression levels in STAS‐positive tumors. Analysis of more cases will help determine the relationship between the morphological and molecular properties of STAS. Among gene mutations, epidermal growth factor receptor (EGFR) is the most actively discussed mutation in the presence of STAS presence. Lee et al.^16^ reported that STAS‐positive tumors were associated with wild‐type *EGFR*, and there were reports suggesting a similar association.^9^, ^10^ Conversely, the association between *EGFR* expression and STAS status could not be established in other studies.^13^, ^14^ In view of these findings and those from another recent report that showed the association between *EGFR* mutation and STAS‐positivity,^18^ there is no clear conclusion on the relationship between STAS status and *EGFR* expression; it is therefore necessary to conduct further research to establish this relationship. To assess the possible role of another gene mutation, Kadota et al.^20^ focused on recent findings that suggest that anaplastic lymphoma kinase (ALK) rearrangement is correlated with specific histological features, such as the cribriform pattern in lung adenocarcinoma.^54^, ^55^ The authors showed that tumors with *ALK* rearrangement tended to have higher STAS‐positivity. Kim et al.^14^ and Lee et al.^16^ reported similar results on *ALK* rearrangements and STAS status. Thus, there are many reports that recognize the association between *ALK* and STAS. Other reports^9^, ^18^ have examined the relationship between v‐raf murine sarcoma viral oncogene homolog B1 (*BRAF*), the Kirsten rat sarcoma viral oncogene homolog (*KRAS*), human epidermal growth factor receptor type2 (HER2), and STAS; however, no significant difference was detected on multivariate analysis, and no definitive conclusion was made on these associations. During the examination of programmed cell death‐1 ligand (PD‐L1) as a key element in the tumor microenvironment and a target of immunotherapy, Toyokawa et al.^13^, ^15^ reported that there is no association between *PD‐L1* expression and the presence of STAS; in addition, no report has shown an association between STAS and *PD‐L1* to date. Little is known regarding the occurrence of STAS; it is expected that further information will be available as research on the molecular biological properties of STAS progresses.

## FUTURE PERSPECTIVES

Based on the general knowledge on cancer biology, STAS‐positive tumor cells need a variety of conditions to move away from the main tumor and survive migration though the air spaces. For STAS, is necessary that: (i) tumor cells are easily separated from the main tumor, (ii) tumor cells survive in remote places (nourished by the surrounding tissues), and (iii) tumor cells are capable of escaping the immune response. Using three‐dimensional histological, immunohistochemical, and multiplex immunofluorescence analyses, Yagi et al.^49^ reported on the survival and growth of STAS; they suggested that STAS detached from the main tumor, migrated through air spaces and reattached to the alveolar walls through vessel co‐option. However, there is no strong biological evidence that STAS‐positive cancer cells can survive in the air space and form metastatic foci away from the main tumor. Masai et al.^30^ reported that cancer cells had more difficulty in colonizing the surface of epithelial tissue than that of mesenchymal tissue. Although STAS‐positive status is clearly a poor prognostic factor in terms of OS and RFS, the current notion that accurate evaluation of STAS is difficult, may be resolved by elucidating the molecular mechanisms of STAS. We hope that the molecular mechanisms of STAS will be elucidated in the near future, and that the concept of STAS will be established more firmly; this will in turn help evaluate lung cancer prognosis accurately, and contribute to the selection of appropriate treatment.

In conclusion, STAS is a poor prognostic factor for recurrence and survival in all histological types of lung cancer, and its presence or absence is likely to have a significant impact on prognosis and treatment for this disease. However, the molecular mechanisms of STAS remain unclear. Further evidence is needed to optimize STAS classification and treatment decisions in STAS‐positive patients.

## CONFLICT OF INTEREST

The authors have no potential conflicts of interest to disclose.
